# Assessing antibiotic residue presence in Turkey meat: insights from a four-box method analysis

**DOI:** 10.1186/s12866-025-03936-2

**Published:** 2025-04-14

**Authors:** Hassna Jaber, Daniel Jesuwenu Ajose, Nabil Fikraoui, Nouhaila Zaazoui, Débora Brito Goulart, Brahim Bourkhiss, Collins Njie Ateba, Mohammed Ouhssine

**Affiliations:** 1 Natural Resources and Sustainable Development Laboratory, Biology Department, Faculty of Sciences, Ibn-Tofail University, B.P 242, Kenitra, Morocco; 2 Food Security and Safety Focus Area, Faculty of Natural and Agricultural Sciences, North-West University, Private Bag X2046, Mmabatho, 2735 South Africa; 3 College of Agriculture and Environmental Science, University of Mohammed VI Polytechnics, Benguerir, Morocco; 4 Laboratory of Microbial Biotechnology and Bioactive Molecules (LM2BM), Faculty of Sciences and Technology, Sidi Mohammed Ben Abdellah University, B.P. 2202, Fes, Morocco; 5 National Animal Disease Center, Virus and Prion Research Unit, United States Department of Agriculture, 1920 Dayton Avenue, Ames, IA 50010 USA; 6 1Animal Plant Production and Agro-industry Laboratory, Department of Biology, Faculty of Science, Ibn-Tofail University, B.P 242, Kenitra, Morocco; 7 School of Biology and Environmental Sciences, Faculty of Agriculture and Natural Sciences, University of Mpumalanga, Mbombela, South Africa

**Keywords:** One Health, Antimicrobial resistance, Turkey meat, Four-box method, Food safety, Poultry farming, Residues

## Abstract

Contemporary poultry farming involves extensive antibiotic use, which could potentially pose health risks to consumers through antibiotic residues in animal-derived food products, especially meat. Recent decisions, particularly the ban on nearly all antibiotic feed additives utilized as growth promoters, have resulted in a decrease in their usage. Nonetheless, their essential role in therapy and their economic value are indisputable. This study evaluated the presence of antibiotic residues in marketed turkey meat using the four-box method. The analyses indicated that, of the 400 samples examined, the overall prevalence of contamination was 65.75%. Among the different types of antibiotics identified in the samples, β-lactam/tetracycline residues were the highest, with a prevalence of 41.44%. The analysis of different sample types revealed significant contamination rates in turkey organs, particularly the liver, with a contamination prevalence of approximately 83.75%, and the wing muscle, 78.75%. Two antibiotic families, β-lactam and tetracycline, were identified in the wing muscle and liver at frequencies of 44.44% and 43.28%, respectively. Regarding cross-contamination, positive samples exhibited contamination concurrently by a specific type of residue, with a notable rate of 58.19%. All analyzed organs exhibited contamination by multiple residue types, with varying contaminants present in different organs. The findings indicated varying detection rates of antibiotic residues in consumed turkey meat. These highlight the excessive use of antibiotics in the poultry industry, increasing consumer exposure to these residues'associated risks. Therefore, it is essential to implement stricter measures and monitoring systems regarding the use of antibiotics in poultry farming.

## Introduction

In Morocco, the poultry industry has been growing significantly and is considered one of the most promising economic sectors in the country. According to a report by the Interprofessional Federation of the Poultry Sector (IFPS) in Morocco in 2023 [33], the poultry industry has recorded a substantial increase in the growth rate. Poultry meat is among the most widely consumed meat products by the Moroccan population, with consumption rising from 16 kg per habitant per year in 2013 to 20.6 kg per habitant per year in 2023 [33]. According to the IFPS, turkey meat production in Morocco started in 2000 with an initial output of 3,000 tons [33]. However, this figure has steadily increased and reached 135,000 tons by 2023 [33]. This increase is due to the relatively low cost of production, the high growth rate, the nutritional value of the meat, and the introduction of new processed products [46].

Nowadays, several veterinary products are used in chicken farming to prevent infections and increase productivity, either with or without veterinarian supervision [52]. Among these products, antibiotics are commonly used for therapeutic purposes, such as treating bacterial infections, or as feed additives to promote growth and enhance feed efficiency [5, 31]. While the use of antibiotics as growth promoters has been banned in the European Union (EU) since 2006 under Regulation No 1831/2003 [24], concerns about antibiotic residues in poultry products remain. This ban was introduced in response to the rising concern of antimicrobial resistance (AMR) caused by the misuse of antibiotics. However, despite the regulatory change, antibiotic residues are still detected in poultry products, particularly in regions where antibiotic use is not strictly regulated or monitored. In Morocco, despite ongoing discussions to reduce antibiotic usage, the regulation of antibiotics as growth promoters remains less stringent compared to the EU [20]. While the EU has implemented a strict ban on the use of antibiotics for growth promotion, Morocco continues to face challenges in enforcing similar regulations [36]. This regulatory gap allows for continued antibiotic use in poultry farming, raising significant concerns about AMR and the safety of poultry products [47].

The presence of antimicrobial residues poses risks to human health, producing immediate toxic or long-term effects in consumers, including but not limited to hypersensitivity reactions and mutagenic and carcinogenic effects [10]. Additionally, the consumption of tainted meat can transmit antibiotic resistance genes from animals, such as turkey, to humans, raising public health concerns [6, 7]. Experimental, epidemiological, and molecular data indicate an association between the use of antimicrobials and the emergence of resistant bacterial strains in animals and their spread to humans, notably through the food chain [35, 38]. The presence of antibiotics in animal food products arises from inadequate adherence to withdrawal periods and the absence of regulations on the maximum authorized residue limit, thus posing a significant risk to public health [34]. The regulation of turkey meat in Morocco is insufficient to ensure compliance with stringent food safety standards observed in developed countries. Public regulatory efforts, such as Morocco's Food Safety Law (Act No. 28–07) and the establishment of the National Food Safety Agency (ONSSA), have begun addressing food safety on a broader scale. However, specific and enforced guidelines for turkey meat production and processing are lacking [17]. Therefore, developing efficient techniques for identifying and measuring antibiotic residues is imperative. The French Food Safety Agency created the four-box method, a standardized microbiological technique for identifying antibiotic residues of the four main antibiotic families Beta-lactamin/tetracycline, Sulfanomides, Aminosides et Beta-lactamin/macrolides [30]. The method, developed by the Laboratory for Studies and Research on Veterinary Drugs and Disinfectants (FFSA, LARMVD) at the Fougère site, is applicable to the muscles of slaughtered animals, as well as to the livers and muscles of palmipedes and poultry [26]. Given the ongoing use of antibiotics in poultry farming in Morocco and the relatively weaker regulatory framework regarding antibiotic residues in poultry products, it is hypothesized that turkey meat marketed in Kenitra City contains detectable levels of antibiotic residues. Furthermore, the study assumes that the four-plate test method can effectively identify and quantify these residues, thereby shedding light on potential public health risks associated with antibiotic use in local poultry farming.

## Materials and methods

### Biological material

Four hundred turkey meat samples, divided into thigh (*n* = 80), upper thigh (*n* = 80), breast (*n* = 80), wing (*n* = 80), and liver (*n* = 80), were purchased at random from various selling points in Kenitra city, Morocco. The selling points were representative of the five most popular districts of the city. Four selling points were randomly chosen in each district, resulting in a total of 20 samples per week. The samples were collected between January and May 2019. This approach led to the collection of 400 turkey meat samples. A minimum weight of 30 g was measured. Each sample was placed in a sterilized bag, sealed, and coded. The samples were later transported in a cooler box to the laboratory and stored at − 20 °C until they were analyzed according to the methods described by Okombe et al. [42].

### Test bacteria strains

Pure strains of *Bacillus subtilis* ATCC 6633 and *Micrococcus luteus* ATCC 14452 were used. The spore suspension of the former, which yields non-reproducible results, was used instead of its vegetative form [40].

### Antibiotics

The antibiotics used in this test were in the form of prepared solutions. Trimethoprim was specifically incorporated into ASS Agar at a pH of 7.4, after sterilization, while the media were maintained in a liquid state at 44 °C in a Bain-marie. This adjustment was crucial as it improved the sensitivity of the test, particularly for the detection of sulphonamide residues, as previously described in El-Youbi et al. [22]. In addition, other antibiotics such as penicillin, sulphadimerazine, dehydrostreptomycin, and erythromycin (Oxoid, Thermo Fisher Scientific, Basingstoke, UK) were included as controls. These controls ensured the accuracy and reliability of the test results by providing reference points for measuring the efficacy of trimethoprim and the detection of sulphonamide residues.

### Culture media

Findey and Field (F-F) and Tryptone soy agar (TSA) (Oxoid, Thermo Fisher Scientific, Basingstoke, UK) culture media were used to revive the bacterial strains, while the Agar Test Culture Medium (pH 6.0), Antibiotic Sulphonamide Sensitivity Test Agar (Agar ASS) Culture Medium (pH 7.4), and Agar Test Culture Medium (pH 8.0) (Oxoid, Thermo Fisher Scientific, Basingstoke, UK) were used in sample analysis.

### Analysis method

The four-plate method is a microbiological method that detect antibiotic residues in a sample without specifying their identity. It is primarily suitable for examining muscles from slaughtered animals and poultry, as well as muscles and livers from fatty palmipeds, but not for kidney samples [22]. This technique involves two main stages. First, a solid nutrient medium is inoculated with a microorganism sensitive to the antibiotics under investigation in Petri dishes. Then, a frozen slice of muscle is placed on the surface of the inoculated medium, followed by incubation at the optimal growth temperature for the test microorganism. This process allows any antibiotic residues in positive samples to diffuse into the medium, resulting in the formation of a zone of inhibition around the sample. To execute this method *B*. *subtilis* was utilized at three different pH levels: 6.0, 7.4, and 8.0, while *M*. *luteus* was used at pH 8.0. These microorganisms served as indicators for antibiotic residues, facilitating the detection process within the specified pH ranges (Table 1).

**Table 1:** Antibiotic classes sought as a function of microorganisms, medium pH, and control solution

Antibiotic classes	Microorganism	Added antibiotic	Medium/pH	Control antibiotics
β-lactam/Tetracycline	*Bacillus subtilis*	-	Agar Test/6.0	Penicillin G
Sulfonamides		Trimethoprim	ASS Agar/7.4	Sulfadimerazine
Aminoglycosides		-	Agar Test/8.0	Dihydrostreptomycin
β-lactam/Macrolides	*Micrococcus luteus*	-	Agar Test/8.0	Erythromycin

### Antibiotic preparation

The preparation of antibiotic solutions was conducted according to the method described by the French Food Safety Agency [26].

A trimethoprim stock solution was prepared by dissolving 50 mg of trimethoprim (Sigma-Aldrich, Taufkirchen, Germany) in 5 mL of 5% acetic acid and adjusting the volume to 500 mL with water in a volumetric flask. This stock solution was diluted to achieve a final concentration of 0.005 mg/mL.

Control solutions were prepared to verify the operating conditions for the four plates. Stock solutions for the antibiotics were prepared as follows:

- **Penicillin G sodium** (Sigma-Aldrich, Taufkirchen, Germany): A stock solution of 61 mg was diluted in 100 mL of water to achieve a final concentration of 0.2 mg/mL.
- **Sulfadimerazine sodium** (Sigma-Aldrich, Taufkirchen, Germany): A stock solution of 50 mg was diluted in 50 mL of 0.1 N NaOH to achieve a final concentration of 0.02 mg/mL.
- **Erythromycin sodium** (Sigma-Aldrich, Taufkirchen, Germany): A stock solution of 54 mg was successively diluted in 50 mL of a methanol–water mixture to reach a final concentration of 0.25 µg/mL.
- **Dihydrostreptomycin sulfate** (Sigma-Aldrich, Taufkirchen, Germany): A stock solution of 64 mg was diluted in 50 mL of water to reach a working concentration of 5 µg/mL.

### Preparation of strains

The preparation of *Bacillus subtilis* and *Micrococcus luteus* followed standardized protocols for reviving lyophilized bacterial strains, as described by Appala et al. [13]. For *B. subtilis*, the lyophilized pure strain was rehydrated in 2 mL of physiological water and inoculated onto Tryptic Soy Agar (TSA) using a sterile cotton swab. The culture was incubated at 30 °C for 24 h, followed by three successive transfers onto TSA slants, with incubation under the same conditions. To harvest the bacteria, glass beads (*n *= 10) and 2 mL of physiological water were added to the slant tube, and the resulting suspension was spread over the surface of F medium poured into Roux dishes (200 mL). The culture was incubated at 30 °C for at least 7 days to promote sporulation. The spores were collected using glass beads and 50 mL of physiological water, washed three times with physiological water, centrifuged at 2000 rpm for 5 min, and the supernatant removed. The final pellet was dissolved in 45 mL of water, transferred to Eppendorf tubes, and enumerated via successive dilutions (up to 10⁻1⁰) on TSA plates incubated at 30 °C for 24 h. The dilution that yielded a spore concentration of 5 × 104 CFU/mL was used for further testing.

For *M. luteus*, the lyophilized strain was rehydrated in 2 mL of 0.85% NaCl and inoculated onto TSA plates, incubated at 37 °C for 24 h. Three successive transfers onto TSA slants were performed, followed by incubation at 37 °C for 24 h. The culture was harvested using glass beads and 2 mL of 0.85% NaCl, then spread over 200 mL of TSA in Roux dishes and incubated at 37 °C for 24 h. The culture was harvested using glass beads and 2 mL of 0.85% NaCl, then spread over 200 mL of TSA in Roux dishes and incubated at 37 °C for 24 h. The bacteria were gathered using glass beads and treated the same way as *B*. *subtilis*. They were then enumerated until the concentration reached 5 × 10^4^ CFU/mL.

#### Preparation of plates

Four types of agar plates were prepared to evaluate the bacterial strains under different conditions. For dishes 1, 2, and 3, *Bacillus subtilis* spore suspension was diluted to achieve a final concentration of ~ 5 × 10^4^ spores per mL of medium. Dish 1 contained an agar test medium at pH 6, dish 2 used Antibiotic Sulphonamide Sensitivity Test Agar (ASS) at pH 7.4 supplemented with trimethoprim solution (0.005 mg/mL) at 1% v/v, and dish 3 contained an agar test medium at pH 8. In each case, 5 mL of the inoculated medium was distributed per Petri dish. For dish 4, the same procedure as dish 3 was followed, except *M. luteus* was used instead of *B. subtilis*.

### Sample processing

Samples were removed from the freezer and placed on a stainless-steel tray. The surface of the muscle was scraped to remove impurities. Frozen cylindrical meat segments 2 mm high and 8 mm in diameter (8 disks for each sample) were cut out using a cookie cutter. Using a pair of forceps, a filter paper disc was placed in the center of each prepared Petri dish, containing 10 µL of control solution on the disc. Finally, the prepared dishes were placed in an incubator at 30 °C and incubated for at least 18 h. The same procedure was applied to pH 7.4 plates. However, the control antibiotic was replaced with sulfadimerazine. Similarly for plates at pH 8 inoculated with *B. subtilis*, the control antibiotic was replaced with dihydrostreptomycin. For plates at pH 8 inoculated with *M. luteus*, an erythromycin disc impregnated at a concentration of 10 µL was placed in the plates and then subjected to incubation conditions at 37 °C for at least 18 h.

#### Reading and interpretation of results

At the end of incubation, discs impregnated with the control solution should show an annular zone of inhibition between 4- and 7 mm. Meat samples with annular zones of inhibition of at least 2 mm were considered positive. If one of the two results obtained was negative and the other was positive, it was considered negative.

#### Statistical analysis

Data was analyzed for the rate of incidence. The statistical study was performed using Microsoft Excel (version 2021). A *p*-value ≤ 0.05 was considered significant.

## Results and discussion

### Total contamination of turkey meat samples analyzed

**Fig. 1:**
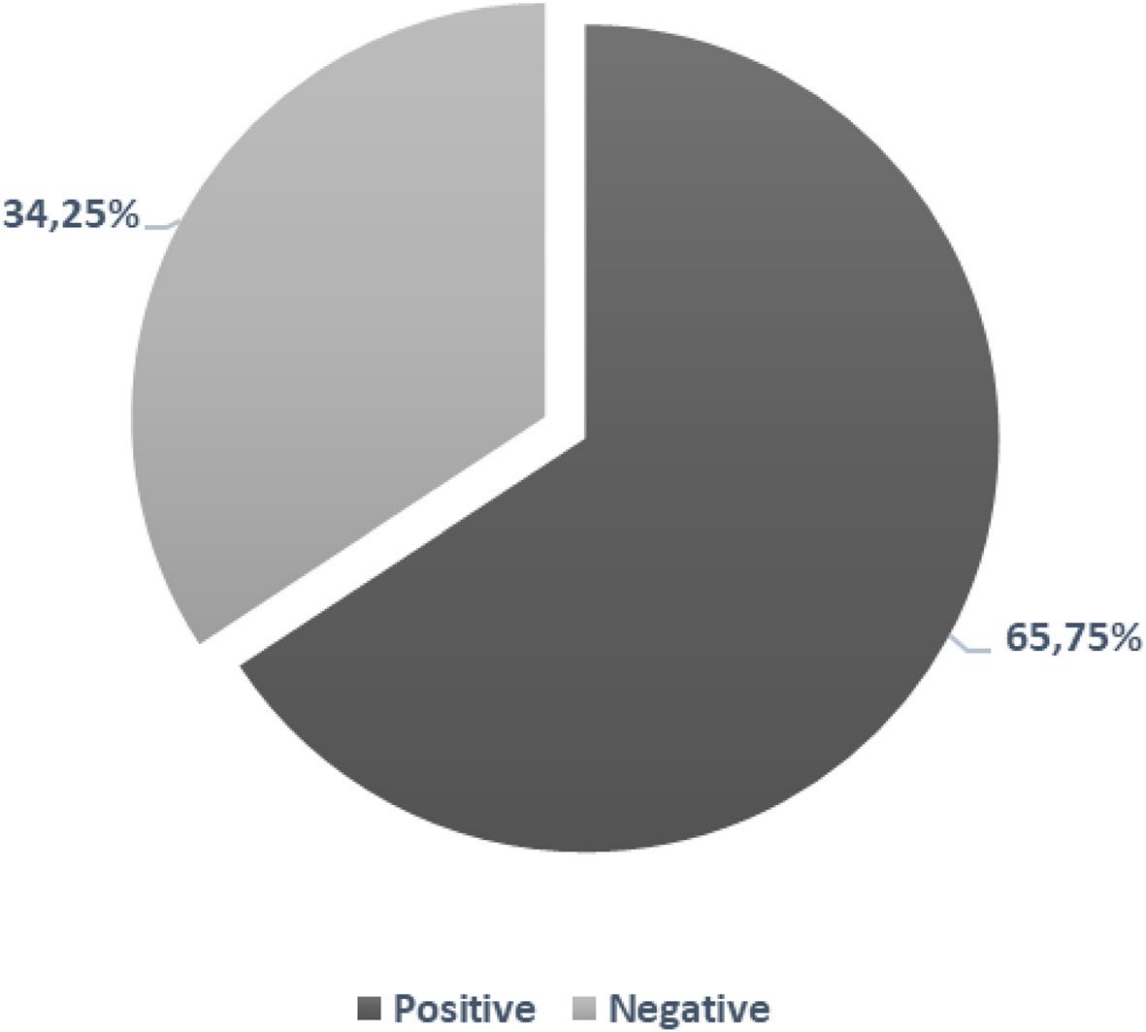
Incidence of antibiotic residue contamination in turkey meat

Detection of antibiotic residues in turkey meat purchased at the point of sale revealed that at least one antibiotic was detected in 263 out of 400 tested samples, representing a prevalence of 65.75% (Fig. 1).

The contamination of food animal origin by veterinary drug residues is a significant public health concern due to its potential to cause allergic reactions, toxicity, and contribute to AMR. Additionally, it undermines compliance with international food safety standards, leading to trade restrictions and economic losses [39]. Several studies have documented the prevalence of antibiotic residues in food products, highlighting the scope of this issue [14, 55]. Indeed, El-Youbi et al. [22] and Al-Mashhadany et al. [11] observed antibiotic residue positive sample rates of 27.45% in chicken meat and 11.10% in poultry meat in Morocco and Iraq, respectively. In Algeria, a study reported the presence of residues with rates of 86.2% in poultry meat [49]. Similarly, Okombe et al. [43] found a contamination rate of 10.40% in poultry meat in Congo, while Ezenduka et al. [25] reported a percentage of residue-positive in poultry samples of 11% in Nigeria. A study carried out in Sudan by Hind et al. [32] showed a positivity rate of 27% in poultry meat. These variations in the reported contamination rates show how important it is to understand the underlying factors that cause antibiotic contamination, such as the microorganism's selective pressure and the rate of antibiotic exposure.

Possible differences in antibiotic detection rates between this study and others could be attributed to sample collection and detection methods'sensitivity. A study by Ahmed et al. [4] has shown that advanced methods and frequent sampling generally result in higher detection rates. Additionally, the strictness of antibiotic rules and veterinary supervision also impacts residue levels [41]. Intensive poultry farming practices, which often involve frequent antibiotic use, further explain higher contamination rates [29]. Understanding these factors is crucial for interpreting and comparing study results accurately [48].

### Distribution of different antibiotic families in the samples analyzed

**Fig. 2:**
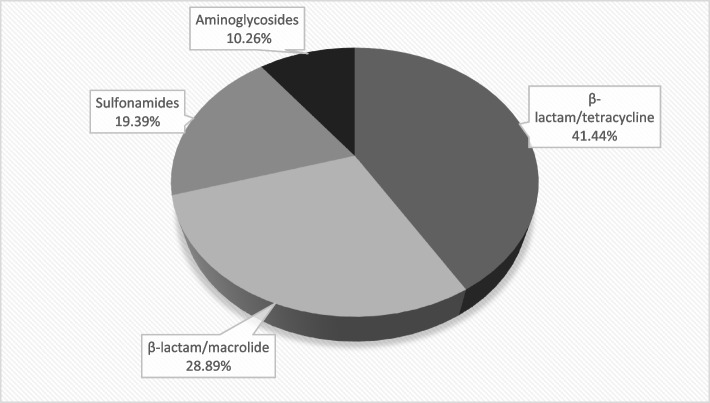
Distribution of antibiotic residues found in turkey meat

According to the types of antibiotics detected in this study and given the results obtained, remarkable contamination of turkey meat by the different residues analyzed was observed (Fig. 2).

The current study revealed significant contamination of turkey meat by antibiotic residues, with β-lactam/tetracyclines being the most prevalent at 41.44%, followed by β-lactam/macrolides (28.89%), sulfonamides (19.39%), and aminoglycosides (10.26%). These findings are consistent with earlier studies. Hakem et al. [30] reported a high contamination rate of 64.83% for β-lactam/tetracycline in turkey meat in Algeria. Similarly, Al-Ghamdi et al. [8] revealed a significant tetracycline rate of 65% in turkey meat in Saudi Arabia, followed by the identification of sulfonamides in all meat samples from different organs. Compared to the current investigation, other studies showed a reduced rate of β-lactam/tetracycline contamination in turkey meat. For example, El-Youbi et al. [22] reported the presence of β-lactam/tetracyclines in 9.80% of the samples analyzed, and the total absence of β-lactams/tetracyclines in turkey meat was reported in studies conducted in Iran [28] and Egypt [1]. The sulfonamide class exhibited a contamination rate of 19.39% in the current study. The study conducted in eastern Morocco by El-Youbi et al. [22] revealed a low percentage of sulfonamides of 9.80% compared to the current study. Studies done out in Algeria by Hakem et al*.* [30] and Ramdane [49] showed sulfonamide percentages of 36.29% and 18.75%, respectively. Of all the antibiotic residues examined in this investigation, the aminoglycoside contamination rate in turkey meat was the lowest, at 10.26%. Compared with the current study, El-Youbi et al. [22] and Hakem et al*.* [30] found rates of aminoglycoside contamination in poultry meat of 5.39% and 13.71%, respectively.

### Antibiotic residue contamination of turkey organ muscles

The contamination rate of turkey organ muscles showed significant incidence rates (Table 2). The liver had the highest contamination rate (83.75%), whereas the breast muscle had the lowest (45%). β-lactam/tetracycline presented the highest contamination rate among all antibiotics included in the study (Table 2).

**Table 2:** Contamination rates of turkey organ muscles by antibiotics tested in the study

Samples	Percentage of positive samples (%)	Antibiotics families			
		β -lactams/Tetracycline (%)	β-lactams/Macrolides (%)	Sulfonamides (%)	Aminoglycosides (%)
Thigh muscle (n = 80)	53.75	41.86	25.58	20.23	11.62
Upper thigh muscle (*n* = 80)	67.50	38.88	31.48	20.37	9.25
Wing muscle (*n* = 80)	78.75	44.44	28.57	19.46	9.52
Breast muscle (*n* = 80)	45.00	36.11	30.55	16.66	8.33
*Liver* (*n* = 80)	83.75	43.28	28.35	17.19	10.44

Several studies have reported the presence of antibiotic residue in turkey meat [15, 23, 37]. Prevalence of colistin resistance in *Escherichia coli* in eastern Turkey and genomic characterization of an *mcr- 1* positive strain from retail chicken meat [3]. Shareef et al. [51] highlighted the predominance of oxytetracycline (28%) and sulfadiazine (24%), mainly in liver organs, muscle, and thigh muscle, while Al-Mashhadany et al. [11] reported 17.80% for liver, 6.70% for breast, and 8.90% for thigh. A study conducted in Egypt examined the presence of residual sipramycin in treated chicken tissues. However, the highest concentration of residues was observed in the liver (40%), and the lowest was found in gizzard and muscle (10%) samples [12]. According to another study conducted in Iraq, the contamination rates were 6.7% in breast muscle 8.9% in thigh muscle, and 17.8% in liver [11]. Surprisingly, Sattar et al. [50] reported varying levels of antibiotic residues in the liver, kidney, and thigh. The highest levels were found in the liver with various antibiotics, including tetracycline (48%), ciprofloxacin (44%), enrofloxacin (40%), and amoxicillin (42%), and the lowest levels were found in the breast muscle (tetracycline − 12%, ciprofloxacin − 15%, enrofloxacin − 9%, and amoxicillin − 11%). Importantly, the liver has the highest contamination rate of antibiotic residues due to its central role in drug metabolism and detoxification. The liver is constantly exposed to high concentrations of drugs as the primary organ for the selective uptake, metabolism, and excretion of drugs. The liver becomes especially susceptible to contamination and hepatocellular damage because of exposure to drugs, which process is potentialized by enzyme systems including the cytochrome P450 family [21].

### Multiple contaminations

**Fig. 3:**
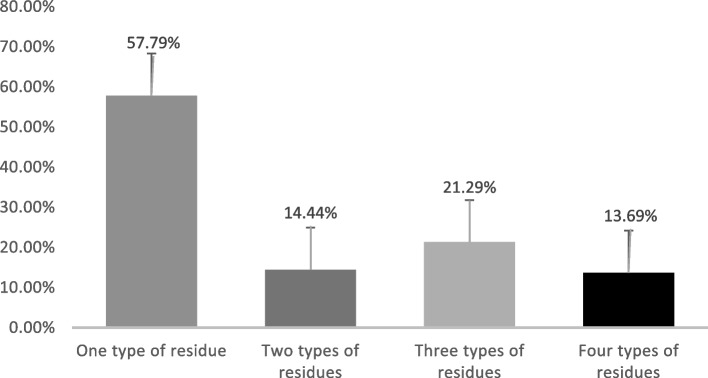
Multiple contaminations of positive samples

Several samples were contaminated by more than one type of antibiotic (Fig. 3). In terms of cross-contamination, 57,79% of positive samples were contaminated by one antibiotic, while 13.69% were contaminated by four antibiotics.

**Fig. 4:**
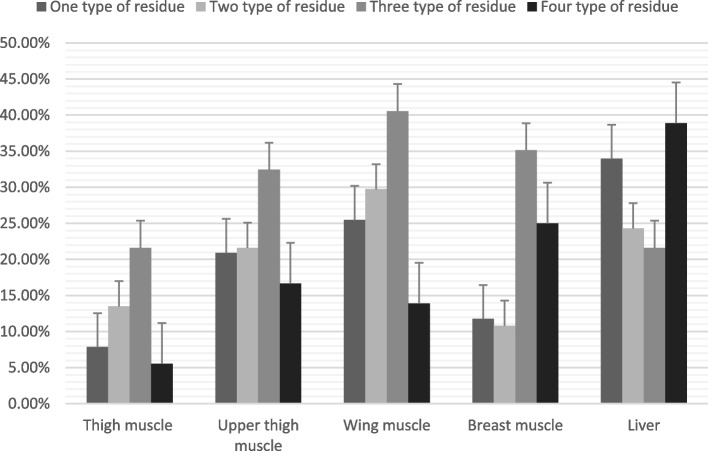
Multiple contaminations of different turkey organs

The highest incidence rate of multiple contaminations was detected in the liver. Wing muscle samples demonstrated contamination by three types of antibiotics at a 40.54% rate, while the liver revealed four types of antibiotic contaminants at a 38.89% rate (Fig. 4).

Compared to the findings of the current study, Okombe et al. [42] found that all samples were contaminated by three types of residues, whereas the percentage of contamination of poultry liver by two types of residues was 75%. All samples tested positive for several antibiotics simultaneously, suggesting that animals had received different antibiotics, either for therapeutic purposes (antibiotic combination therapy) or as growth promoters. According to Tyers and Wright [53], antibiotic combinations are prescribed to broaden the spectrum of activity. Paul et al*.* [45], on the other hand, consider the combination of several antibiotics to be an outcome to avoid therapeutic failure. Importantly, the high percentage of positive cases might be due to non-compliance with waiting periods [19, 27]. As a result, the unofficial use of antibiotics as growth promoters is highly suspected, despite the ban on this practice. Given that most poultry farmers are not professionals, and they do not master the application of basic antibiotic therapeutic rules, the misuse of antibiotics benefits the development of an unfavourable environment for poultry, leading to the emergence of various pathologies, including AMR [9]. The One Health approach is vital for addressing the issue of AMR, as it emphasizes the interconnectedness of human, animal, and environmental health [18, 44]. In Morocco, this approach has become increasingly important due to the widespread use of antibiotics in agriculture and the resulting risks to human and animal health [16]. Despite regulations, the overuse of antibiotics in poultry farming contributes to the development of resistant pathogens that can be transmitted through food, direct contact, and environmental contamination. Additionally, improper disposal of antibiotic-laden waste can lead to the spread of resistant bacteria in water sources and the broader environment [2, 47].

By incorporating One Health principles, Morocco could take a more integrated approach to combat AMR, involving collaboration across human health, veterinary, and environmental sectors [54]. This approach would allow for better regulation, surveillance, and prevention strategies to address the root causes of AMR, ensuring that both public health and agricultural practices are aligned in the fight against resistance. Antibiotic resistance is a worldwide problem that is causing increasing public health concerns, and it is now recognized as a crucial One Health issue. When thinking about what could be done to stop or lessen this problem, it is very important to understand the complex factors that have led to the rise of antibiotic-resistant bacteria. This means that AMR measures taken (or not taken) in one division may affect others [6, 7].

## Conclusion

The current study reveals significant contamination of turkey meat with antibiotic residues, with varying incidence rates depending on the antibiotic families and organs examined. Multiple contamination was widespread across all tested samples, highlighting the excessive and unregulated use of antibiotics in agriculture, particularly in livestock farming. This misuse continues to pose risks to human health, contributing to AMR and the persistence of residues in food. Effective regulation of these residues is crucial for ensuring public health, protecting trade compliance, and ensuring the production of high-quality agricultural products.

A One Health approach is essential for addressing AMR, as it connects human, animal, and environmental health. All stakeholders in the poultry industry must collaborate to mitigate the risks of AMR and antibiotic residue contamination. Future research should focus on improving detection methods, exploring sustainable alternatives to antibiotic use in agriculture, and assessing the long-term effects of exposure on human health. Additionally, evaluating current regulatory frameworks and strengthening enforcement could significantly improve global food safety standards.

A limitation of this study is its focus on specific antibiotic families and turkey organs, which may not represent the broader range of contamination in other livestock. Future studies should address economic and regulatory factors influencing antibiotic use in agriculture, providing a more comprehensive understanding of the issue.

## Data Availability

The datasets generated and/or analysed during the current study are available.
